# External validation of the European risk assessment tool for chronic cardio-metabolic disorders in a Middle Eastern population

**DOI:** 10.1186/s12967-020-02434-5

**Published:** 2020-07-02

**Authors:** Samaneh Asgari, Fatemeh Moosaie, Davood Khalili, Fereidoun Azizi, Farzad Hadaegh

**Affiliations:** 1 Prevention of Metabolic Disorders Research Center, Research Institute for Endocrine Sciences, Shahid Beheshti University of Medical Sciences, Tehran, Iran; 2 Tehran University of Medical Sciences, Tehran, Iran; 3 Department of Biostatistics and Epidemiology, Research Institute for Endocrine Sciences, Shahid Beheshti University of Medical Sciences, Tehran, Iran; 4 Endocrine Research Center, Research Institute for Endocrine Sciences, Shahid Beheshti University of Medical Sciences, Tehran, Iran

**Keywords:** Risk assessment, External validation, Cardiovascular disease, Diabetes mellitus, type 2, Chronic kidney disease

## Abstract

**Background:**

High burden of chronic cardio-metabolic disorders including type 2 diabetes mellitus (T2DM), chronic kidney disease (CKD), and cardiovascular disease (CVD) have been reported in the Middle East and North Africa region. We aimed to externally validate a non-laboratory risk assessment tool for the prediction of the chronic cardio-metabolic disorders in the Iranian population.

**Methods:**

The predictors included age, body mass index, waist circumference, use of antihypertensive medications, current smoking, and family history of cardiovascular disease and/or diabetes. For external validation of the model in the Tehran lipids and glucose study (TLGS), the Area under the curve (AUC) and the Hosmer–Lemeshow (HL) goodness of fit test were performed for discrimination and calibration, respectively.

**Results:**

Among 1310 men and 1960 women aged 28–85 years, 29.5% and 47.4% experienced chronic cardio-metabolic disorders during the 6 and 9-year follow-up, respectively. The model showed acceptable discrimination, with an AUC of 0.72 (95% CI 0.69–0.75) for men and 0.73 (95% CI 0.71–0.76) for women. The calibration of the model was good for both genders (min HL P = 0.5). Considering separate outcomes, AUC was highest for CKD (0.76 (95% CI 0.72–0.79)) and lowest for T2DM (0.65 (95% CI 0.61–0.69)), in men. As for women, AUC was highest for CVD (0.82 (95% CI 0.78–0.86)) and lowest for T2DM (0.69 (95% CI 0.66–0.73)). The 9-year follow-up demonstrated almost similar performances compared to the 6-year follow-up. Using Cox regression in place of logistic multivariable analysis, model’s discrimination and calibration were reduced for prediction of chronic cardio-metabolic disorders; the issue which had more effect on the prediction of incident CKD among women. Moreover, adding data of educational levels and marital status did not improve, the discrimination and calibration in the enhanced model.

**Conclusion:**

This model showed acceptable discrimination and good calibration for risk prediction of chronic cardio-metabolic disorders in short and long-term follow-up in the Iranian population.

## Background

Chronic cardio-metabolic disorders including type 2 diabetes mellitus (T2DM), chronic kidney disease (CKD), and cardiovascular disease (CVD) have a considerably higher overall disability-adjusted life years (DALYs) in the Middle East and North Africa region (MENA) compared to their global estimates. In the past three decades, the prevalence of T2DM has increased 1.5–2 times in the MENA making it the region with the second-highest T2DM prevalence in 2017 globally [1, 2]. Iran is the second-largest country in the MENA with the increasing prevalence of obesity, T2DM, CKD, and hypertension leading to CVD. Moreover, the incidence density rate of T2DM, CKD, and CVD were 10.6, 21.5, and 10.5 respectively per 1000 person-year over more than 10 years follow-up in an Iranian population [3, 4]. Age-standardized mortality from non-communicable diseases (NCDs) among populations aged 30–70 was 346.1 per 100,000 population in 2016 [5]. Programs for screening and primary prevention have been reported in Iran but so far scarcely implemented [6, 7].

Incident T2DM, CKD, and CVD share many risk factors including age, sex, obesity, smoking, high blood pressure, and sedentary lifestyle. Diabetes is a risk factor for both CKD and CVD [3, 4, 8]. To date, various models have been proposed for the prediction of T2DM [9, 10], CKD [11], and CVD [10, 12–15] separately. Most of the previous models were comprised of non-laboratory measures. Sattar N. et al. [10] suggest in a recent article that the best approach for screening cardio-metabolic disease is to start from non-laboratory measures in the primary phase and employ laboratory measures only for the high-risk group of individuals. In 2012 a model comprising of non-laboratory measures was suggested by Alssema et al. [16] for the 7-year risk prediction of combined endpoints (i.e. T2DM, CKD, and CVD) in the Dutch population. It revealed good discrimination between low- and high-risk populations for the combined outcomes. This prediction tool is now implemented into Dutch guidelines for general practitioners [17]. This model was validated in Australia in 2018 which revealed good discrimination and poor calibration [18]. The development and validation of this model have been performed predominantly in the Europoid population and it might not be transferable to the other ethnicities. This prediction model is comprised of non-laboratory measures which make it a cost-worthy tool for screening and primary prevention of chronic cardio-metabolic disorders especially in countries of the MENA with limited healthcare facilities. Therefore, in the current study, we aimed to externally validate the risk prediction tool for chronic cardio-metabolic disorders in the Iranian population. Moreover, we also examined the validity of this model to predict chronic cardio-metabolic disorders during an extended follow-up period of 9 years.

## Methods

### Study population

Tehran Lipid and Glucose Study (TLGS) is a community-based prospective cohort study conducted on an Iranian urban population in Tehran. The study aims to determine the prevalence and incidence of non-communicable diseases and related risk factors among individuals aged ≥ 3 years and promote a healthy lifestyle and programs for the prevention of NCDs. The study has been established in two phases including the first (1999–2001; n = 15,005) and the second (2001–2005; n = 3550) and is designed to keep on for at least 20 years on the triennial basis. The design and methodology of the TLGS study have been reported elsewhere [19]. Since the detail of the data regarding the cardiovascular status at the recruitment time was available from the phase II, the current study was designed on 7490 individuals aged 28–85 years who participated in the second phase of the TLGS study (phase I = 5716 and phase II = 1774). From this number, we excluded those with prevalent CVD (i.e. participants with a history of myocardial infarction, angioplasty, coronary artery bypass graft (CABG) or stroke, (n = 546)), prevalent T2DM defined as self-reported use of diabetes-lowering medication (n = 856) and prevalent end-stage renal disease (ESRD) defined by estimated Glomerular Filtration Rate (eGFR) < 15 mL/min/1.73 m^2^ (n = 1). After excluding those with missing data at baseline for creatinine (Cr), fasting plasma glucose (FPG), 2-hour post-challenge plasma glucose (2 h-PCG), body mass index (BMI), waist circumference (WC) and smoking status (n = 1864, considering overlap features between missing values) as well as participants with missing data during follow-up on Cr (n = 32), FPG, 2 h-PCG (n = 718) and CVD status (n = 203), 3270 individuals were eligible for the current study during 6-year follow-up until March 2011. In line with the risk assessment tool, no one died from non-cardiovascular causes during the follow-up period.

To investigate the long-term effect of the risk assessment tool for prediction of chronic cardio-metabolic disorders, from a total of 4223 individuals, we excluded prevalent cases of CVD, T2DM, and ESRD and those with missing data on covariates using the above approach. 3240 individuals remained for the analysis during 9-year follow-up until March 2018 (Additional file 1: Figure S1). This study was approved by the Institutional Review Board (IRB) of the Research Institute for Endocrine Sciences (RIES), Shahid Beheshti University of Medical Sciences, Tehran, Iran, and all participants provided written informed consent.

### Clinical and laboratory measurements

Information on demographic data, family history of premature CVD and T2DM, current smoking status, and medication history were obtained by a trained interviewer using a standard questionnaire. Details for anthropometric measurements including height, weight, and WC are reported elsewhere [19]. A blood sample was taken from all study participants between 7:00 and 9:00 AM after 12 to 14 h overnight fasting. More detail for laboratory measurements including FPG, 2 h-PCG, and serum creatinine was addressed previously [19].

### Definition of variables

BMI was calculated as weight (kg) divided by height (m^2^). A positive family history of premature CVD for the study participant was considered as having previously diagnosed CVD in first-degree male and female relatives aged < 55 and  < 65 years, respectively. The current smoker was defined as who smokes cigarettes daily or occasionally.

#### Outcomes

- Type 2 diabetes
  T2DM was defined as FPG ≥ 7 mmol/L, 2 h-PCG ≥ 11.1 mmol/L or use of anti-diabetic medications.
- Chronic kidney disease
  CKD was defined as eGFR < 60 mL/min/1.73 m^2^, provided by the Modification of Diet in Renal Disease (MDRD) [20, 21].
- Cardiovascular disease
  According to the previously published article about CVD outcomes in the TLGS cohort [22, 23], each participant is followed-up for any medical event leading to hospitalization during the previous year by telephone call. They were asked for any medical conditions by a trained nurse and later, a trained physician collected complementary data regarding that event during a home visit and by the acquisition of data from medical files. The collected data were then evaluated by an outcome assessment committee consisting of an internist, endocrinologist, cardiologist, epidemiologist, and other experts, if required, to assign a specific outcome for every event. In the current study, CHD events included cases of definite and probable MI, unstable angina, angiographic proven CHD, heart failure, and CHD death. Stroke was also defined as a definite or possible stroke or transient ischemic attack. Finally, CVD was clarified as a composite measure of any CHD events, stroke, or cerebrovascular death.
- Chronic cardio-metabolic disorders
  Chronic cardio-metabolic disorders was defined as the diagnosis of either T2DM, CKD or CVD during the follow-up period.

### Risk tool for chronic cardio-metabolic disease

To evaluate the chronic cardio-metabolic disorders outcome, the risk tool was developed on 6780 Dutch men and women (aged 28–85 years) based on three population-based cohorts: the Rotterdam study(n = 4018), the Hoorn study (n = 627) and the Prevention of Renal and Vascular End-stage Disease (PREVEND; n = 2135) [16]. The sex-stratified model including age, BMI, WC, use of antihypertensive medications, current smoking, parent and/or sibling with MI or stroke (age < 65 years), and parent and/or sibling with diabetes were developed using logistic regression (Additional file 2: Table S1). The 7-year risk of chronic cardio-metabolic disorders was calculated for each subject according to the original risk assessment tool recommended by Alssema et al. [16] for each TLGS men and women.

### Statistical analysis

Baseline characteristics of respondents (those with complete data) and non-respondents (those with missing data of covariates or loss to follow-up) were expressed as mean (standard deviation) and number (%) for categorical variables. For covariates with a skewed distribution, the median (interquartile range: IQR) was reported. A comparison of baseline characteristics between men and women was done by the Student’s *t* test for normally distributed continuous variables, Maan -Whitney u test for skewed variables, and the Chi squared test for categorical variables. To evaluate the external validity of the risk equation, Area under the curve (AUC) and Hosmer–Lemeshow Chi square were applied to determine the discrimination and calibration of these predictor models, respectively. According to the Hosmer et al. [24] criteria, the AUCs 0.5–0.7, 0.70–80, 0.80–0.90, and ≥ 0.90 indicated poor, acceptable, excellent, and outstanding discrimination, respectively. Bootstrapping method with 1000 replications was used to estimate the uncertainty interval [25, 26].To show the calibration in detail, the observed risk was plotted versus the mean of predicted probabilities using the calibration belt Stata module [27]. Besides, the observed to an expected ratio (O/E) for the chronic cardio-metabolic disorders outcome was calculated; ratio < 1 indicated overestimation, and > 1 indicated underestimation of the risk.

We also recalibrated the risk assessment tool for the TLGS cohort characteristics by adjusting the intercept of the model; the same predictors with the same regression coefficients of the original model were fixed while the intercept was estimated as the free parameters [28]. The clinical performance of the validated model was evaluated using the same scoring point as defined by Alssema et al. [16]. The non-laboratory risk score was calculated by summing the risk points over the defined variables for both men and women (Additional file 2: Table S1). The cut-off point in the TLGS data was assessed by the maximum value of the Youden index (sensitivity + specificity-1) in each gender. Besides, the frequency of the high-risk population was calculated according to the 2016 national Iranian censuses. Using the above statistical approach, we repeated our data analysis for those participants with a 9-year follow-up. To compare the discrimination measurement of the risk assessment tool with other available non-invasive prediction models for the CVD outcome, we used the Gaziano et al. [13] risk score. We also assessed whether adding educational levels and marital status, two important social factors that previously showed moderate association with incident T2DM and CKD among Iranian population [29, 30], would improve the discrimination or calibration. Furthermore, to avoid complete case analysis bias, we accounted for missing information on the baseline variables (outcomes were not imputed) by using single imputation methods. The missing values were estimated using multivariable regression models [31]. A sensitivity analysis was done by re-estimating the same covariates as Alssema et al. [16] in the validated sample using Cox-proportional hazard regression. To check for the discriminative power of the models, Harrell’s C index (95% CI) was used. Statistical analysis was performed using STATA version 14 (StataCorp LP, College Station, Texas), statistical software. *p *< 0.05 were considered as statistically significant.

## Results

### Baseline characteristics

The study population consisted of 1310 men and 1960 women at baseline with a mean (SD) age of 47.1 (12.8) and 45.3 (11.3) years, respectively. The baseline characteristics of men and women are shown in Table 1. There were significant differences between men and women; men were older and had a higher level of WC and higher frequencies of being a current smoker, whereas women had a higher level of BMI and higher frequencies of using antihypertensive medications and positive family history of CVD. The comparison of the baseline characteristics of the respondents vs. non-respondents is shown in Additional file 3: Table S2.

**Table 1:** baseline characteristics and incidence of the outcome: Tehran Lipid and glucose study

	Men (N = 1310)	Women (N = 1960)	*p*
Age (years)			< 0.001
< 45	672 (51.3)	1065 (54.34)	
≤ 45 to > 50	165 (12.6)	258 (13.16)	
≤ 50 to > 55	121 (9.24)	215 (10.97)	
≤ 55 to > 60	108 (8.24)	177 (9.03)	
≤ 60 to > 65	73 (5.57)	129 (6.58)	
≤ 65 to > 70	89 (6.79)	70 (3.57)	
≤ 70 to > 75	60 (4.58)	35 (1.79)	
≤ 75 to > 85	22 (1.68)	11 (0.56)	
Total, mean (SD)	47.1 (12.8)	45.3 (11.3)	< 0.001
Body mass index (kg/m^2^)			< 0.001
< 25	440 (33.59)	394 (20.1)	
≤ 25 to < 30	615 (46.95)	845 (43.11)	
≥ 30	255 (19.47)	721 (36.79)	
Total, mean (SD)	26.8 (4.0)	28.9 (4.6)	< 0.001
Waist circumference (cm)			< 0.001
Men < 94 and women < 80	576 (43.97)	307 (15.66)	
Men ≥ 94 to < 102 and women ≥ 80 to < 88	410 (31.3)	436 (22.24)	
Men ≥ 102 and women ≥ 88	324 (24.73)	1217 (62.09)	
Total, mean (SD)	94.9 (10.4)	91.5 (11.6)	< 0.001
Use of antihypertensive medications (yes)	42 (3.21)	148 (7.55)	< 0.001
Current smoking (yes)	406 (30.99)	110 (5.61)	< 0.001
Family history diabetes (yes)	440 (33.6)	624 (31.8)	0.29
Family history premature CVD (yes)	231 (17.63)	392 (20.0)	0.01
Incidence of chronic cardio-metabolic disorders	387 (29.54)	929 (47.4)	< 0.001
T2DM			
De novo detected T2DM at baseline	64 (4.94)	90 (4.66)	0.71
Incidence of T2DM	105 (8.01)	149 (7.6)	0.62
CKD			
De novo detected CKD at baseline	53 (4.0)	239 (12.2)	< 0.0001
Incidence of CKD	175 (13.3)	547 (27.9)	< 0.0001
Incidence of CVD	73 (5.57)	49 (2.5)	< 0.001
Follow-up duration, median (IQR)	6.26 (5.65–7.0)	6.22 (5.56–7.0)	< 0.001

Data are shown as mean (SD) for continues and number (%) for categorical covariates; IQR: Interquartile range; CVD: cardiovascular disease; T2DM: Type 2 diabetes; CKD; chronic kidney disease; CVD: cardiovascular disease

During the median (IQR) follow-up of 6.2 years (5.6–7.0), the cumulative incidence of chronic cardio-metabolic disorders among the whole population was 1316 (40.2%), the corresponding values for men and women were 387 (29.54%) and 929 (47.4%), respectively. The cumulative incidence of T2DM, CKD, and CVD were 12.9%, 17.4%, and 5.57% among men, compared to 12.19%, 40.1%, and 2.5% among women, respectively. Of all incident T2DM (n = 408), 154 had undiagnosed T2DM at baseline; of total individuals with incident CKD (n = 1014), 292 had an eGFR ranged between 15 and 60 mL/min/1.73 m^2^ at baseline.

### Model performance

According to Table 2, the combined risk score showed acceptable discrimination for incident chronic cardio-metabolic disorders in the TLGS study, with AUC (95% CI) of 0.72 (0.69–0.75) in men and 0.73 (0.71–0.76) in women. Restricting CVD events, we found similar discrimination to chronic cardio-metabolic disorders in men, but significantly higher discrimination value in women; the AUC (95% CI) was 0.82 (0.78–0.86) that indicates excellent discrimination. Considering CKD outcome, the discrimination value was 0.76 (95% CI 0.72–0.79) in men and 0.71 (95% CI 0.69–0.74) in women. Poor discrimination was shown for T2DM events; AUC was 0.65 (95% CI 0.61–0.69) in men and 0.69 (95% CI 0.66–0.73) in women. A receiver operating characteristic curve for men and women is presented in Additional file 4: Figure S2.

**Table 2:** Model performance for 6-year and 9-year follow-up: Tehran lipid and glucose study

	Chronic cardio-metabolic disorders	T2DM	CKD	CVD
Men				
AUC (95% CI) *				
Original Follow-up 6y	0.72 (0.69–0.75)	0.65 (0.61–0.69)	0.76 (0.72–0.79)	0.73 (0.68–0.79)
Intercept adjusted Follow-up 6y	0.72 (0.69–0.75)	0.65 (0.60–0.69)	0.76 (0.72–0.79)	0.73 (0.68–0.79)
Original Follow-up 9y	0.71 (0.68–0.74)	0.66 (0.62–0.69)	0.71 (0.67–0.74)	0.71 (0.66–0.75)
Intercept adjusted Follow-up 9y	0.71 (0.68–0.74)	0.66 (0.62–0.70)	0.71 (0.67–0.74)	0.71 (0.66–0.75)
HL test				
Original Follow-up 6y	6.87 (*p* value = 0.55)	7.71 (p-value = 0.46)	12.82 (p-value = 0.12)	18 (p-value = 0.02)
Intercept adjusted Follow-up 6y	7.21 (p-value = 0.51)	7.34 (p-value = 0.5)	13.53 (p-value = 0.09)	18.3 (p-value = 0.02)
Original Follow-up 9y	10.7 (p-value = 0.22)	8.77 (p-value = 0.36)	13.21 (p-value = 0.1)	13.3 (p-value = 0.1)
Intercept adjusted Follow-up 9y	11.0 (p-value = 0.2)	8.7 (p-value = 0.37)	12.54 (p-value = 0.13)	14.2 (p-value = 0.08)
Women				
AUC (95% CI)^a^				
Original Follow-up 6y	0.73 (0.71–0.76)	0.69 (0.66–0.73)	0.71 (0.69–0.74)	0.82 (0.78–0.86)
Intercept adjusted Follow-up 6y	0.73 (0.71–0.75)	0.69 (0.66–0.73)	0.71 (0.69–0.73)	0.82 (0.78–0.86)
Original Follow-up 9y	0.72 (0.70–0.75)	0.68 (0.65–0.72)	0.70 (0.68–0.72)	0.81 (0.76–0.85)
Intercept adjusted Follow-up 9y	0.72 (0.70–0.75)	0.68 (0.65–0.71)	0.70 (0.68–0.73)	0.81 (0.77–0.85)
HL test				
Original Follow-up 6y	5.62 (p-value = 0.69)	36.3 (p-value < 0.001)	10.1 (p-value = 0.26)	14.9 (p-value = 0.06)
Intercept adjusted Follow-up 6y	5.62 (p-value = 0.69)	36.3 (p-value < 0.001)	10.1 (p-value = 0.26)	14.9 (p-value = 0.06)
Original Follow-up 9y	6.4 (p-value = 0.6)	35.2 (p-value < 0.001)	8.19 (p-value = 0.41)	11.2 (p-value = 0.2)
Intercept adjusted Follow-up 9y	6.4 (p-value = 0.6)	35.1 (p-value < 0.001)	8.19 (p-value = 0.41)	11.2 (p-value = 0.2)

^a^With 1000 Bootstrapping

The recalibrated intercept for follow-up 6 years was estimated -1.51 for men and -2.71 for women; the recalibrated intercept for follow-up 9 years was estimated -0.71 for men and -1.89 for women

The total sample size was 1314 for men (composite outcome = 589, T2DM = 252, CKD = 378, CVD = 120) and 1926 for women (composite outcome = 1125, T2DM = 315, CKD = 981, CVD = 80)

AUC: area under the curve; CI confidence interval; HL; Hosmer–Lemeshow test; T2DM: type 2 diabetes; CKD: chronic kidney disease; CVD: cardiovascular disease

The secondary analysis during the median (IQR) 9.2 years (8.7–10.2) follow-up, demonstrated almost similar discrimination and calibration for both genders compared with the 6-year follow-up (Table 2).

**Fig. 1:**
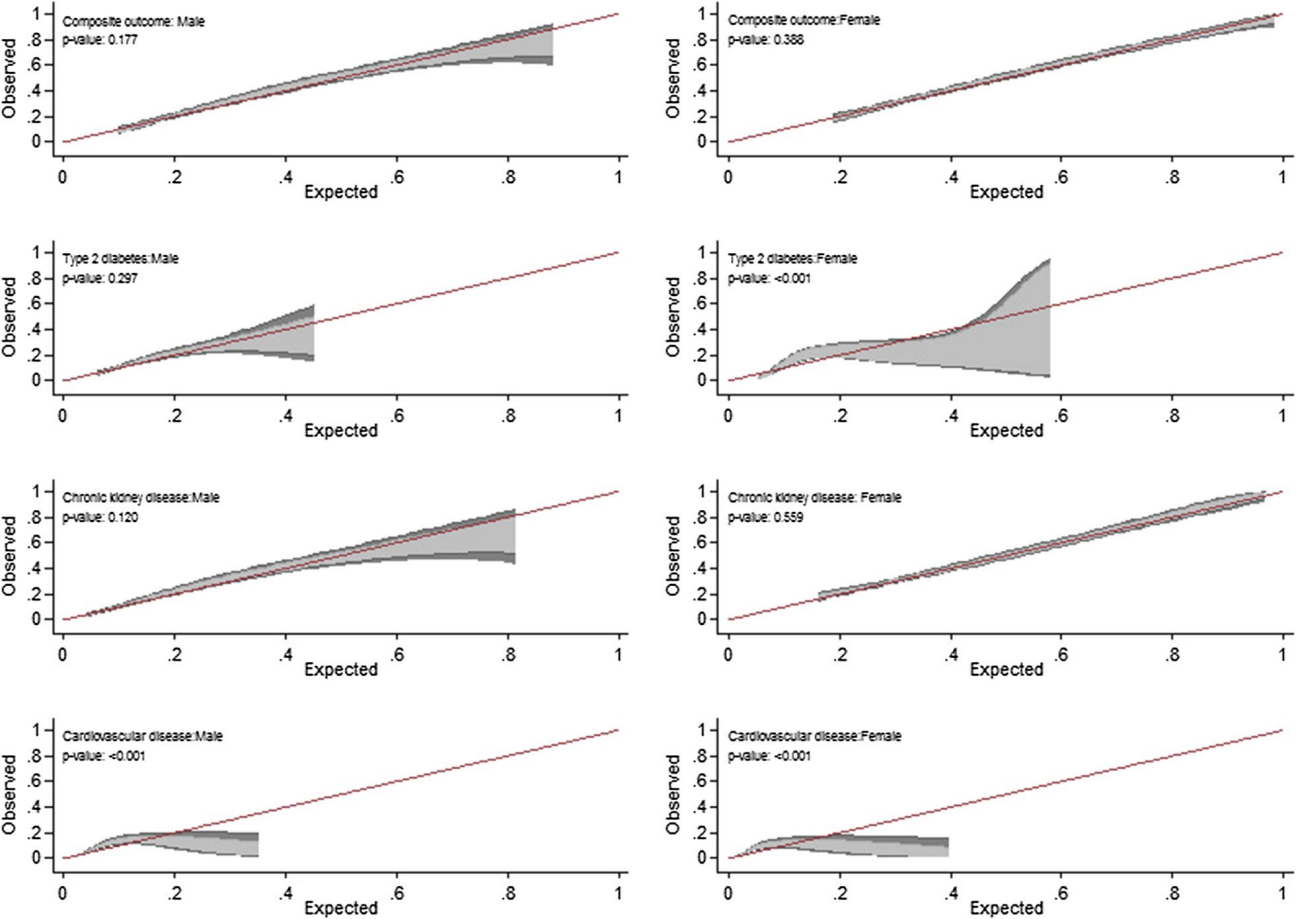
Calibration belt plot of the risk of a prediction tool for T2DM, CKD, CVD, and Chronic cardio-metabolic disorders outcomes among men and women separately. The Solid line indicates the bisector line(perfect calibration). The light-gray area defines an 80% confidence level. The dark-gray area defines a 95% confidence level. A likelihood-ratio test was used for evaluating the hypothesis of good calibration (p > 0.05). Composite outcome: Chronic cardio-metabolic disorders; T2DM: type 2 diabetes; CKD: chronic kidney disease; CVD: cardiovascular disease

The Hosmer–Lemeshow goodness-of-fit test showed good calibration for men (χ^2^ = 6.87, *p *= 0.55) and women (χ^2^ = 5.62, *p *= 0.69) for incident chronic cardio-metabolic disorders. Focusing on each of chronic cardio-metabolic disorders outcomes, the calibration was poor for men with CVD events (HL test: *p *= 0.02) and poor for women with incident T2DM (HL test: *p *= <0.001). The observed-expected plot was shown in Fig. 1. The hypothesis of the good calibration was not rejected for incident chronic cardio-metabolic disorders in men and women. However, considering T2DM (among women) and CVD (for both genders), the hypothesis of the good calibration was rejected. Moreover, recalibration with adjusting the TLGS study intercept did not improve the model goodness-of-fit; HL tests were significant regarding T2DM for women and CVD for men. Also, the AUC showed similar discrimination compared with the original model (Table 2). The O/E ratio for the combined cardio-metabolic disease was almost 1 for both men and women.

When implicating the score threshold of ≥ 35 from the Alssema, et al. [16] among our population, 21.8% of men were detected as high-risk population and yielded a sensitivity of 42.9%, and specificity 87.0%; this cut-off point detected 20.8% of women as a high-risk group and yielded a sensitivity of 35.4%, and specificity of 92.3%. Hence, considering the low sensitivity of the derived cut-off point for prediction of chronic cardio-metabolic disorders in our population, we calculated cut-off points Using Youden’s index [32]. The results showed that the cut-off point ≥ 25 for men and ≥ 19 for women during 6-year follow-up has the highest Youden’s index. These cut-points detected 43.9% of men and 50.6% of women as high-risk for chronic cardio-metabolic disorders; yielded the sensitivity of 63.8%, and the specificity of 70.9% for men and the sensitivity of 66.7%, and the specificity of 70.0% for women (Table 3). The estimated values for 9-year follow-up are reported in Additional file 5: Table S3.

**Table 3:** The clinical performance of the risk assessment tool for 6-year prediction of chronic cardio-metabolic disorders and each of the separate diseases: Tehran Lipid and glucose study

	Chronic cardio-metabolic disorders		T2DM		CKD		CVD	
	Validation	Developed	Validation	Developed	Validation	Developed	Validation	Developed
Men (N = 1310)								
Number of events at score category	261	166	102	68	173	120	59	37
Number of high-risk populations	575	286	575	286	575	286	575	286
Sensitivity, %	63.8	42.9	57.4	40.2	72.7	52.6	79.4	50.7
Specificity, %	70.9	87	63.3	80.9	67.6	84.7	63	79.9
Women (N = 1960)								
Number of events at score category	646	329	189	85	542	304	49	32
Number of high-risk populations	992	408	992	408	992	408	992	408
Sensitivity, %	66.7	35.4	75.3	35.6	66.8	38.7	95.9	65.3
Specificity, %	70.0	92.3	56.5	81.2	65.6	91.1	53.8	80.3

T2DM: Type 2 diabetes; CKD; chronic kidney disease; CVD: cardiovascular disease

The validation cut-off point: ≥ 25 for men and ≥ 19 for women

The developed cut-off point: ≥ 35 for men and women

The number of urban population of Tehran aged 28–85 year according to the Iranian census 2016: 7,214,301 men and 7,223,540 women

Number at risk in Tehran according to the validation cut-off point: 3,166,583 men and 3,655,996 women

Number at risk in Tehran according to the developed cut-off point: 1,587,146 men and 1,516,943 women

### Additional analysis

The AUC (95% CI) of non-laboratory based risk-score for predicting incident CVD, proposed by Gaziano et al. [13], was 0.78 (0.73–0.82) for men and 0.82 (0.78–0.87) for women. Moreover, the AUC (95% CI) of non-laboratory based risk-score for predicting incident chronic cardio-metabolic disorders, proposed by Gaziano et al. [13], was 0.72 (0.69–0.75) for men and 0.75 (0.72–0.77) for women.

We also repeated our analysis using Iranian WC cut-point (≥ 95 cm for both genders) [33] and the results remained unchanged (Additional file 6: Table S4).

We examined the additional value of the educational levels and marital status in the univariable analysis and they were significant predictors of defined outcomes for both men and women;both lost their significance after adjusting the chronic cardio-metabolic disorders risk score into the model. Considering the educational level, discrimination and calibration did not improve in the enhanced model (Additional file 7: Table S5). Moreover, considering marital status in the enhanced model, it only improved the calibration performance of the incident CVD; the discrimination remained almost unchanged (Additional file 8: Table S6).

Based on the results of the imputed data set (Additional file 9: Table S7) the discrimination and calibration remained essentially unchanged.

In the sensitivity analysis using Cox regression in place of logistic multivariable analysis, model’s discrimination and calibration were reduced for prediction of chronic cardio-metabolic disorders; the issue which had more effect on the prediction of incident CKD among women. However, some improvements were observed in the discrimination and the calibration of the model for prediction of incident T2DM (Additional file 10: Table S8).

## Discussion

The current study is the second global and the first non-Europoid external validation of a previously developed, non-laboratory based 7-year risk prediction tool for chronic cardio-metabolic disorders. This model showed acceptable discrimination and good calibration for 6- and 9-year risk prediction of chronic cardio-metabolic disorders among the metropolitan city of Tehran. In women, the model performed best for discriminating CVD followed by CKD and among men, for both CVD and CKD. The model performed worst for predicting T2DM in both genders during both follow-up periods. Moreover, the performance of the model remained the same even with the updated cutoff values considering the Iranian ethnicity.

Generally compared with the development data [16], our study population is younger, more obese (general or central), with a higher frequency of family history of T2DM, and lower frequency of premature CVD.

**Fig. 2:**
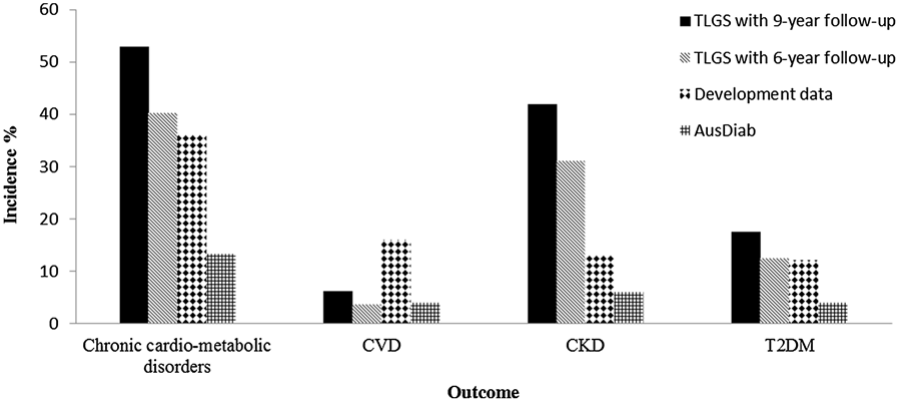
The incidence of chronic cardiometabolic disease in the development data (Netherlands), AusDiab (Australia), and TLGS (Tehran Lipid and glucose study). T2DM: type 2 diabetes; CKD: chronic kidney disease; CVD: cardiovascular disease

The model showed an acceptable discriminative performance in the TLGS population despite the lower AUC levels (0.72 and 0.73 for men and women, respectively) compared to the development data (AUC of 0.80 and 0.82 for men and women, respectively) [16] and the AusDiab study (AUC of 0.78 and 0.80 for men and women, respectively) [18]. This difference might be explained by the difference in the discrimination for the specific NCDs groups despite the higher incidence of chronic cardio-metabolic disorders (40.2%) compared to the development (36.0%) and AusDiab data (13.3%) (Fig. 2). Moreover, in the current study, we reported the high prevalence of newly diagnosed T2DM and CKD (i.e. those with eGFR 15 to 60 mL/min/1.73 m^2^) among Iranian population at baseline compared to the development (4.6% and 7.2%, respectively) [16] and AusDiab data (3.7% and 11.2%, respectively) [34, 35]. An efficient risk prediction model requires a series of assumptions to eliminate the potential presence of reverse causality [36]. We believe that the high prevalence of newly diagnosed T2DM among the TLGS population at the baseline caused reverse causality that might have affected obesity indices, leading to lower performance of the model in the prediction of T2DM.

Focusing on cut-off points for prediction of chronic cardio-metabolic disorders, the Alssema et al. [16] demonstrated a cut-off point between 35 and 40 with high sensitivity (75 to 85% for men and 83 to 90% for women) and moderate specificity (55 to 66% for men and 49 to 62% for women). Considering the derived score thresholds in our data, the sensitivity levels decreased significantly for predicting chronic cardio-metabolic disorders (Max 42.9%). Since, cut-off point selection depends on the implemented population, considering a trade-off between sensitivity and specificity, score ≥ 25 for men and ≥ 19 for women indicates an acceptable value of sensitivity (63.8% for men and 66.7% for women) and specificity (70.9% for men and 70.0% for women) for predicting 6-year chronic cardio-metabolic disorders in our poplation.

According to the medical university reports [37–39], there are 77 health centers in Tehran, each of which covers more than 50,000 people [40] living in the 22 districts of city. Based on the results, of total, 6,722,079 Tehranian aged 28–85 years old (according to the 2016 Iran census) will be classified as high-risk, which requires further investigations. Accordingly,the number of existing centers are not enough and it is recommended to increase this number.

The incidence of CVD was lower in the TLGS population compared to the development and AusDiab data (Fig. 2). There are several previously developed models comprising non-laboratory measures for the prediction of CVD only [13, 41]. One of which is a model introduced by Gaziano et al. [13] for the prediction of CVD. The aforementioned model revealed no significant difference in CVD discrimination compared to our adjusted model. When comparing the performance of the current model with the non-laboratory INTERHEART risk score (AUC = 0.74 (0.70 to 0.78)) in the Middle East population, our model showed good calibration in the 9-year follow-up, better discrimination in women and the same discriminative performance in men; Despite not including T2DM as a major risk factor in chronic cardio-metabolic disorders model [41]. Although CVD showed less contribution to the composite outcome, the model revealed the best CVD discrimination for women and the second-best discriminative performance for CVD in men for both follow-up periods. Other models for prediction of CVD showed the same gender difference as the current model. Framingham CVD risk score is one of the models also validated in Iran. The results were in line with ours and showed higher discrimination in women compared to men [15]. The model showed a good calibration for CVD for both genders during the 9-year follow-up. This could be explained by the time-dependent course of CVD progression leading to a higher rate of CVD incidence in the long-term follow-up.

The incidence of CKD was higher in the TLGS population compared to the development and AusDiab data (Fig. 2). There are several previously developed models comprising non-laboratory measures for the prediction of CKD [42]. CKD had the most contribution to the composite chronic cardio-metabolic disorders outcome. This could be due to the presence of multiple major risk factors of CKD in the current model including age, hypertension, and smoking. The inclusion of laboratory measures could increase the predictive power of the model as it has been addressed in a meta-analysis (C-statistic probability = 0.845) [11]. Considering that only non-laboratory measures were included in the model and eGFR was absent as a major predictor of CKD, an AUC of about 0.76 in men and 0.71 in women is acceptable. The model showed the best CKD discrimination for men and the second-best discriminative performance for CKD in women. Calibration was good for the prediction of CKD.

Focusing on T2DM, its incidence was almost similar to the development data but higher than the AusDiab population (Fig. 2). The model showed the worst discriminative performance for T2DM in both men and women. Calibration was good in men but poor in women. Several explanations could be proposed for the poor performance of the model in the prediction of T2DM. Firstly, as mentioned earlier the percentage of newly diagnosed T2DM in the TLGS study was higher compared to the development and AusDiab data [16, 35]; this issue affects the discriminative power of the chronic cardio-metabolic disorders model for incident T2DM. Secondly, during 6-years follow-up we previously found that general adiposity was not an independent risk factor for incident T2DM, however including age, SBP as well as waist to hip ratio(WHR) and waist to height ratio(WHtR) in a non-laboratory model resulted in an AUC of 0.75 (0.72–0.78) [43]. This suggests that replacing BMI or WC with WHtR or WHR could boost the discriminative power of the model for T2DM. Several other studies have also established the higher predictive power of WHtR compared to WC and BMI in the prediction of T2DM [44]. Most of the previously developed prediction tools for T2DM (Finish Diabetes Risk Score (FINDRISK), ADA risk score and AUSDRISK) are indicative of the same discriminative performance as our results; except for AUSDRISK which manifests somehow better discriminative performance in prediction of DM (AUC = 0.787 (0.747–0.787) in TLGS data) [9, 45, 46].

This study had several strengths. Firstly, this is the first study to validate this model in a non-Europoid population especially in the MENA region with a high burden of NCDs. Secondly, we also examined the accuracy of this model in an extended follow-up period. Thirdly, the calculation of ethnicity-based cut-off values and comparing them with the original cutoff values suggested an insensitivity of the model to ethnicity-based cut-off values.

This study had several limitations. Firstly, in the TLGS study, the incident angina pectoris, history of intermittent claudication (using Rose questionnaire), and peripheral intervention was not assessed, so as shown in Fig. 2, the incident CVD events might have been underestimated. However, despite differences in CVD definitions with the original study, the discriminative power of the chronic cardio-metabolic disorders tool for CVD assessment was acceptable. A higher incidence rate of CVD might have resulted in better calibration performance; which was not captured by updating the intercept as shown in Table 2. However, the calibration improved in longer follow-up (9-year) due to a higher number of CVD events. Regarding heart failure, although it was not assessed at the baseline recruitments, it was evaluated as one of the CVD outcomes in the TLGS study [22]. Secondly, men participants, compared to non-respondents were more obese and reported a higher rate of smoking while women participants reported less frequency of smoking and use of anti-hypertensive medications; leading to over- and underestimation of chronic cardio-metabolic disorders among men and women, respectively (Additional file 3: Table S2). Thirdly, as reported in TLGS protocol, age and sex distribution of the population in district No. 13 is representative of the overall urban population of Tehran and Iran at the time of baseline recruitment (Iran National Census, 1996) [19], hence our findings might not be generalizable to the entire population of Iran, especially rural areas. Finally, the values of serum creatinine were not calibrated to the Cleveland Clinic.

The current risk prediction tool is freely available on websites in the Netherlands and is also incorporated into the Dutch guidelines for general practitioners, ‘The Prevention Visit’ [17]. Recent studies have discussed the cost-effectiveness of the cardio-metabolic risk assessment [47, 48]. This model could help differentiate between the high-risk population in need for further risk assessment and those at low risk in the MENA region.

## Conclusion

In conclusion, the previously developed non-invasive 7-year risk prediction tool for chronic cardio-metabolic disorders performed well in regards to discrimination and calibration in a non-Europoid population with a 6- and 9-year follow up. The model performed best for prediction of CVD and CKD in both genders but further workup evaluation is needed for better prediction of T2DM. Results from this study suggest that this model has an acceptable performance in other ethnic groups and for a longer follow-up period. World health organization (WHO) has implemented a prevention program to reduce death from NCD by 25% in the Eastern Mediterranean region by 2025 [49]. This non-laboratory cost-effective tool is especially very beneficial for screening three important NCDs in middle to low-income regions with limited access to health care facilities.

## Supplementary information

**Additional file 1: Figure S1:** Study flowchart. TLGS: Tehran lipids and glucose study; CVD: cardiovascular disease; ESRD: End-Stage Renal Disease; Cr: creatinine; BMI: body mass index; WC; waist circumference; fasting plasma glucose: FPG; 2-hour post-challenge plasma glucose: 2 h-PCG. *No deaths were recorded during follow-up from non-cardiovascular causes.

**Additional file 2: Table S1:** Risk assessment tool regression coefficients for the chronic cardiometabolic disease developed in the Dutch population.

**Additional file 3: Table S2:** Comparing baseline characteristics between Respondents and non- respondents: Tehran Lipid and glucose study.

**Additional file 4: Figure S2:** A Roc curve for chronic cardio-metabolic disorders and each outcome separately during 6-year follow-up in men and women: Tehran lipid and glucose study. ROC: receiver operating characteristic; CVD: cardiovascular disease; CKD: chronic kiney disease; T2DM: type 2 diabetes.

**Additional file 5: Table S3:** The clinical performance of the risk assessment tool for 9-year prediction of chronic cardio-metabolic disorders and each of the separate diseases: : Tehran Lipid and glucose study. T2DM: Type 2 diabetes; CKD; chronic kidney disease; CVD: cardiovascular disease. The validation cut-off point: ≥ 25 for men and ≥ 23 for women. The developed cut-off point: ≥ 35 for men and women. The number of urban population of Tehran aged 28–85 year: 7,214,301 men and 7,223,540 women. Number at risk in Tehran according to the validation cut-off point is 2,904,388 men and 5,823,342 women. Number at risk in Tehran according to the developed cut-off point 1,636,120 men and 1,469,007 women.

**Additional file 6: Table S4:** Model performance for 6-year and 9-year follow-up using the Iranian waist circumference cut-point (≥ 95 cm): Tehran lipid and glucose study. * With 1000 Bootstrapping. The total sample size was 1314 for men (composite outcome = 589, T2DM = 252, CKD = 378, CVD = 120) and 1926 for women (composite outcome = 1125, T2DM = 315, CKD = 981, CVD = 80). AUC: area under the curve; CI confidence interval; HL; Hosmer–Lemeshow test; T2DM: type 2 diabetes; CKD: chronic kidney disease.

**Additional file 7: Table S5.** Model performance for 6-year and 9-year follow-after adjusting for educational levels: Tehran lipid and glucose study. * With 1000 Bootstrapping. The total sample size was 1314 for men (composite outcome = 589, T2DM = 252, CKD = 378, CVD = 120) and 1926 for women (composite outcome = 1125, T2DM = 315, CKD = 981, CVD = 80). AUC: area under the curve; CI confidence interval; HL; Hosmer–Lemeshow test; T2DM: type 2 diabetes; CKD: chronic kidney disease; CVD: cardiovascular disease. The AUC was estimated on the predictive probability after further adjustment with educational levels.

**Additional file 8: Table S6:** Model performance for 6-year and 9-year follow-after adjusting for marital status: Tehran lipid and glucose study. * With 1000 Bootstrapping. The total sample size was 1314 for men (composite outcome = 589, T2DM = 252, CKD = 378, CVD = 120) and 1926 for women (composite outcome = 1125, T2DM = 315, CKD = 981, CVD = 80). AUC: area under the curve; CI confidence interval; HL; Hosmer–Lemeshow test; T2DM: type 2 diabetes; CKD: chronic kidney disease; CVD: cardiovascular disease. The AUC was estimated on the predictive probability after further adjustment with marital status.

**Additional file 9: Table S7.** Model performance for 6-year (n = 4522) and 9-year (n = 4001) after imputation: Tehran lipid and glucose study. * With 1000 Bootstrapping. 6-year follow-up: 1919 for men (composite outcome = 547, T2DM = 313, CKD = 317, CVD = 104) and 2603 for women (composite outcome = 1182, T2DM = 397, CKD = 997, CVD = 67) 1679. 9-year follow-up: for men (composite outcome = 752, T2DM = 235, CKD = 512, CVD = 87) and 1679 for women (composite outcome = 1445, T2DM = 308, CKD = 1259, CVD = 55). AUC: area under the curve; CI confidence interval; HL; Hosmer–Lemeshow test; T2DM: type 2 diabetes; CKD: chronic kidney disease; CVD: cardiovascular disease.

**Additional file 10: Table S8:** Model performance for 6-year and 9-year using Cox-regression: Tehran lipid and glucose study. * With 1000 Bootstrapping. AUC: area under the curve; CI confidence interval; HL; Hosmer–Lemeshow test; T2DM: type 2 diabetes; CKD: chronic kidney disease; CVD: cardiovascular disease.

## Data Availability

The datasets used and/or analyzed during the current study are available from the corresponding author on reasonable request.

## Abbreviations

T2DM: Type 2 diabetes mellitus
CKD: Chronic kidney disease
DALYs: Disability-adjusted life years
MENA: Middle East and North Africa region
NCDs: Non-communicable diseases
TLGS: Tehran Lipid and Glucose Study
CABG: Coronary artery bypass graft
ESRD: End-stage renal disease
eGFR: Estimated Glomerular Filtration Rate
Cr: Creatinine
FPG: Fasting plasma glucose
2 h-PCG: 2-hour post-challenge plasma glucose
BMI: Body mass index
WC: Waist circumference
MDRD: Modification of Diet in Renal Disease
PREVEND: Prevention of Renal and Vascular End-stage Disease
IQR: Interquartile range
AUC: Area under the curve
O/E: Observed to an expected ratio
WHR: Waist to hip ratio
WHtR: Waist to height ratio

## References

[1] Azizi F, Hadaegh F, Hosseinpanah F, Mirmiran P, Amouzegar A, Abdi H (2019). Metabolic health in the Middle East and north Africa. Lancet Diab Endocrinol.

[2] Worldwide trends in diabetes since 1980: a pooled analysis of 751 population-based studies with 44 million participants. Lancet (London, England). 2016;387(10027):1513-30. 10.1016/S0140-6736(16)00618-8 PMC5081106 27061677

[3] Tohidi M, Hasheminia M, Mohebi R, Khalili D, Hosseinpanah F, Yazdani B (2012). Incidence of chronic kidney disease and its risk factors, results of over 10 year follow up in an Iranian cohort. PLoS ONE.

[4] Sardarinia M, Akbarpour S, Lotfaliany M, Bagherzadeh-Khiabani F, Bozorgmanesh M, Sheikholeslami F (2016). Risk factors for incidence of cardiovascular diseases and all-cause mortality in a middle eastern population over a decade follow-up: Tehran Lipid and glucose Study. PLoS ONE.

[5] Danaei G, Farzadfar F, Kelishadi R, Rashidian A, Rouhani OM, Ahmadnia S (2019). Iran in transition. Lancet (London, England)..

[6] Faraji O, Etemad K, Akbari Sari A, Ravaghi H (2015). Policies and programs for prevention and control of diabetes in iran: a document analysis. Global J Health Sci.

[7] Peykari N, Hashemi H, Dinarvand R, Haji-Aghajani M, Malekzadeh R, Sadrolsadat A (2017). National action plan for non-communicable diseases prevention and control in Iran; a response to emerging epidemic. J Diab Metab Disorders..

[8] Arnett DK, Blumenthal RS, Albert MA, Buroker AB, Goldberger ZD, Hahn EJ (2019). 2019 ACC/AHA Guideline on the Primary Prevention of Cardiovascular Disease: a Report of the American College of Cardiology/American Heart Association Task Force on Clinical Practice Guidelines. Circulation.

[9] Abbasi A, Peelen LM, Corpeleijn E, van der Schouw YT, Stolk RP, Spijkerman AMW (2012). Prediction models for risk of developing type 2 diabetes: systematic literature search and independent external validation study. Br Med J.

[10] Sattar N, Gill JMR, Alazawi W (2020). Improving prevention strategies for cardiometabolic disease. Nat Med.

[11] Nelson RG, Grams ME, Ballew SH, Sang Y, Azizi F, Chadban SJ, et al. Development of Risk Prediction Equations for Incident Chronic Kidney Disease. Jama. 2019. 10.1001/jama.2019.17379 PMC6865298 31703124

[12] D’Agostino RB, Vasan RS, Pencina MJ, Wolf PA, Cobain M, Massaro JM (2008). General cardiovascular risk profile for use in primary care: the Framingham Heart Study. Circulation.

[13] Gaziano TA, Young CR, Fitzmaurice G, Atwood S, Gaziano JM (2008). Laboratory-based versus non-laboratory-based method for assessment of cardiovascular disease risk: the NHANES I Follow-up Study cohort. Lancet (London, England)..

[14] Joseph P, Yusuf S, Lee SF, Ibrahim Q, Teo K, Rangarajan S (2018). Prognostic validation of a non-laboratory and a laboratory based cardiovascular disease risk score in multiple regions of the world. Heart.

[15] Khalili D, Hadaegh F, Soori H, Steyerberg EW, Bozorgmanesh M, Azizi F (2012). Clinical usefulness of the framingham cardiovascular risk profile beyond its statistical performance: the tehran lipid and glucose Study. Am J Epidemiol.

[16] Alssema M, Newson RS, Bakker SJ, Stehouwer CD, Heymans MW, Nijpels G (2012). One risk assessment tool for cardiovascular disease, type 2 diabetes, and chronic kidney disease. Diabetes Care.

[17] Dekker JM, Alssema M, Janssen PG, Goudswaard LN (2011). Summary of the practice guideline ‘The Prevention Visit’ from the Dutch College of General Practitioners. Ned Tijdschr Geneeskd.

[18] Rauh SP, Rutters F, van der Heijden AAWA, Luimes T, Alssema M, Heymans MW (2018). External validation of a tool predicting 7-year risk of developing cardiovascular disease, type 2 diabetes or chronic kidney disease. J Gen Intern Med.

[19] Azizi F, Ghanbarian A, Momenan AA, Hadaegh F, Mirmiran P, Hedayati M (2009). Prevention of non-communicable disease in a population in nutrition transition: tehran Lipid and Glucose Study phase II. Trials..

[20] Levey AS, Coresh J, Bolton K, Culleton B, Harvey KS, Ikizler TA, et al. K/DOQI clinical practice guidelines for chronic kidney disease: evaluation, classification, and stratification. American Journal of Kidney Diseases. 2002;39(2 SUPPL. 1). 11904577

[21] Levey A (2000). A simplified equation to predict glomerular filtration rate from serum creatinine. J Am Soc Nephrol.

[22] Kabootari M, Asgari S, Mansournia MA, Khalili D, Valizadeh M, Azizi F (2018). Different weight histories and risk of incident coronary heart disease and stroke: tehran lipid and glucose study. J Am Heart Assoc.

[23] Khalili D, Azizi F, Asgari S, Zadeh-Vakili A, Momenan AA, Ghanbarian A, et al. Outcomes of a longitudinal population-based Cohort Study and pragmatic community trial: Findings from 20 years of the Tehran Lipid and Glucose Study. Int J Endocrinol Metab. 2018;16(4 Suppl). 10.5812/ijem.84748 PMC6289305 30584434

[24] Hosmer DW, Lemeshow S, Sturdivant RX (2013). Applied logistic regression.

[25] Hadaegh F, Asgari S, Bozorgmanesh M, Jeddi S, Azizi F, Ghasemi A (2016). Added value of total serum nitrate/nitrite for prediction of cardiovascular disease in middle east caucasian residents in Tehran. Nitric Oxide.

[26] Pencina MJ, D’Agostino RB, D’Agostino RB, Vasan RS (2008). Evaluating the added predictive ability of a new marker: from area under the ROC curve to reclassification and beyond. Stat Med.

[27] Nattino G, Lemeshow S, Phillips G, Finazzi S, Bertolini G (2017). Assessing the calibration of dichotomous outcome models with the calibration belt. Stata J.

[28] Steyerberg EW (2019). Clinical prediction models.

[29] Derakhshan A, Sardarinia M, Khalili D, Momenan AA, Azizi F, Hadaegh F (2014). Sex specific incidence rates of type 2 diabetes and its risk factors over 9 years of follow-up: Tehran Lipid and Glucose Study. PLoS ONE.

[30] Tohidi M, Hasheminia M, Mohebi R, Khalili D, Hosseinpanah F, Yazdani B (2012). Incidence of chronic kidney disease and its risk factors, results of over 10 year follow up in an Iranian cohort. PLoS ONE.

[31] Van der Heijden GJ, Donders ART, Stijnen T, Moons KG (2006). Imputation of missing values is superior to complete case analysis and the missing-indicator method in multivariable diagnostic research: a clinical example. J Clin Epidemiol.

[32] Perkins NJ, Schisterman EF (2006). The inconsistency of “optimal” cutpoints obtained using two criteria based on the receiver operating characteristic curve. Am J Epidemiol.

[33] Hadaegh F, Zabetian A, Sarbakhsh P, Khalili D, James W, Azizi F (2009). Appropriate cutoff values of anthropometric variables to predict cardiovascular outcomes: 7.6 years follow-up in an Iranian population. Int J Obes.

[34] Chadban SJ, Briganti EM, Kerr PG, Dunstan DW, Welborn TA, Zimmet PZ (2003). Prevalence of kidney damage in Australian adults: the AusDiab kidney study. J Am Soc Nephrol.

[35] Dunstan DW, Zimmet PZ, Welborn TA, De Courten MP, Cameron AJ, Sicree RA (2002). The rising prevalence of diabetes and impaired glucose tolerance: the Australian Diabetes. Obesity and Lifestyle Study. Diabetes care..

[36] Sattar N, Preiss D (2017). Reverse causality in cardiovascular epidemiological research: more common than imagined?. Am Heart Assoc..

[37] Shahid Beheshti University of medical sciences. http://www.sbmu.ac.ir/index.jsp?fkeyid=&siteid=1&pageid=2055. Accessed 14 June 2020.

[38] Tehran University of medical sciences. Health care centers. https://sthn.tums.ac.ir/index.php/%D9%85%D8%B1%D8%A7%DA%A9%D8%B2-%D9%88-%D8%AE%D8%A7%D9%86%D9%87-%D9%87%D8%A7%DB%8C-%D8%A8%D9%87%D8%AF%D8%A7%D8%B4%D8%AA.html. Accessed 14 June 2020.

[39] Iran medical University. https://iums.ac.ir/page/1496/%D9%85%D8%B1%D8%A7%DA%A9%D8%B2-%D9%88-%D8%B4%D8%A8%DA%A9%D9%87-%D9%87%D8%A7%DB%8C-%D8%A8%D9%87%D8%AF%D8%A7%D8%B4%D8%AA%DB%8C-%D8%AF%D8%B1%D9%85%D8%A7%D9%86%DB%8C. Accessed 14 June 2020.

[40] Ministry of Health and Education. http://zaums.ac.ir/21257. Accessed 14 June 2020.

[41] Joseph P, Yusuf S, Lee SF, Ibrahim Q, Teo K, Rangarajan S (2018). Prognostic validation of a non-laboratory and a laboratory based cardiovascular disease risk score in multiple regions of the world. Heart (British Cardiac Society)..

[42] Hippisley-Cox J, Coupland C (2010). Predicting the risk of chronic Kidney Disease in men and women in England and Wales: prospective derivation and external validation of the QKidney Scores. BMC Family Practice..

[43] Bozorgmanesh M, Hadaegh F, Ghaffari S, Harati H, Azizi F (2011). A simple risk score effectively predicted type 2 diabetes in Iranian adult population: population-based cohort study. Eur J Pub Health.

[44] Ashwell M, Gunn P, Gibson S (2012). Waist-to-height ratio is a better screening tool than waist circumference and BMI for adult cardiometabolic risk factors: systematic review and meta-analysis. Obes Rev.

[45] Lotfaliany M, Hadaegh F, Asgari S, Mansournia MA, Azizi F, Oldenburg B (2019). Non-invasive Risk Prediction Models in Identifying Undiagnosed Type 2 Diabetes or Predicting Future Incident Cases in the Iranian Population. Arch Iran Med.

[46] Asgari S, Lotfaliany M, Fahimfar N, Hadaegh F, Azizi F, Khalili D. The external validity and performance of the no-laboratory American Diabetes Association screening tool for identifying undiagnosed type 2 diabetes among the Iranian population. Primary Care Diabetes. 2020. 10.1016/j.pcd.2020.04.001 32522438

[47] Badenbroek IF, Stol DM, Nielen MM, Hollander M, Kraaijenhagen RA, de Wit GA (2014). Design of the INTEGRATE study: effectiveness and cost-effectiveness of a cardiometabolic risk assessment and treatment program integrated in primary care. BMC Family Pract.

[48] Badenbroek IF, Stol DM, Nielen MM, Hollander M, Kraaijenhagen RA, de Wit GA (2016). Erratum to: design of the INTEGRATE study: effectiveness and cost-effectiveness of a cardiometabolic risk assessment and treatment program integrated in primary care. BMC Family Pract.

[49] World Health Organization, Regional Office for Eastern Mediterranean 2020. http://www.emro.who.int/entity/ncds/index.html. Accessed 3 May 2020.

