# Supplementary figures and images for: External validation of the European risk assessment tool for chronic cardio-metabolic disorders in a Middle Eastern population
Source: J Transl Med. 2020 Jul 2;18:267. doi: 10.1186/s12967-020-02434-5 (PMC7331242; doi:10.1186/s12967-020-02434-5)

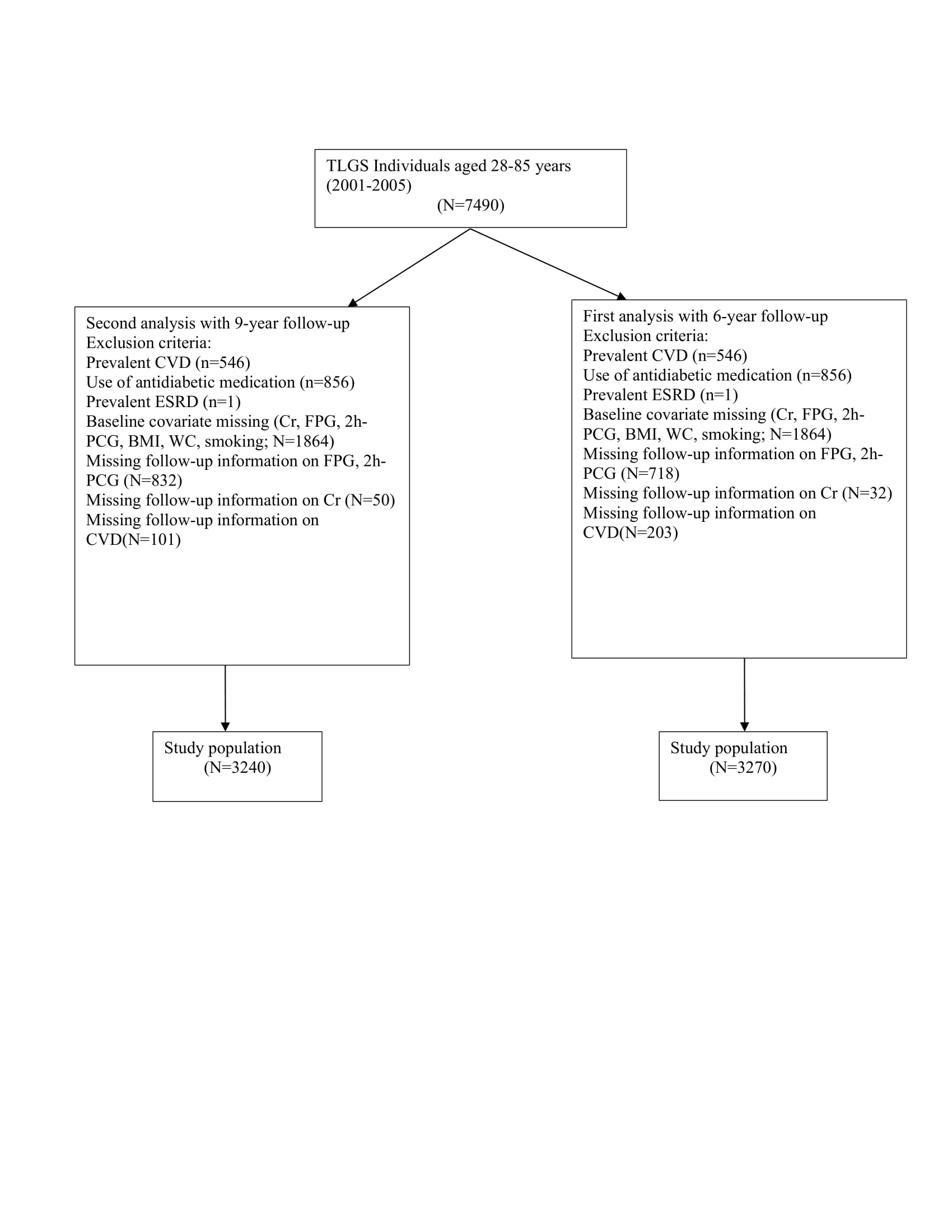

Supplement: Supplementary file 1 — Additional file 1: Figure S1: Study flowchart. TLGS: Tehran lipids and glucose study; CVD: cardiovascular disease; ESRD: End-Stage Renal Disease; Cr: creatinine; BMI: body mass index; WC; waist circumference; fasting plasma glucose: FPG; 2-hour post-challenge plasma glucose: 2 h-PCG. *No deaths were recorded during follow-up from non-cardiovascular causes. [file 12967_2020_2434_MOESM1_ESM.jpg]

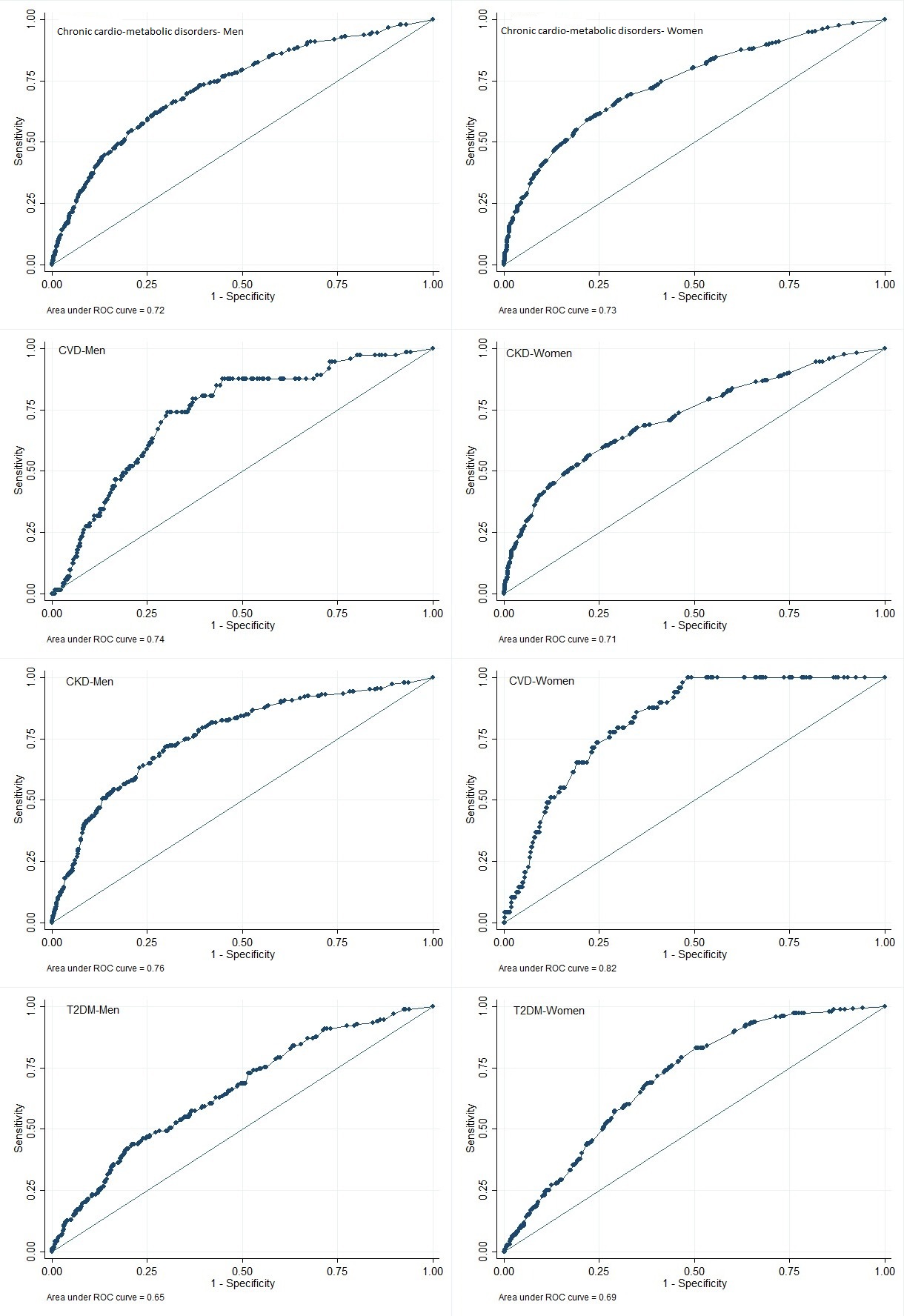

Supplement: Supplementary file 4 — Additional file 4: Figure S2: A Roc curve for chronic cardio-metabolic disorders and each outcome separately during 6-year follow-up in men and women: Tehran lipid and glucose study. ROC: receiver operating characteristic; CVD: cardiovascular disease; CKD: chronic kiney disease; T2DM: type 2 diabetes. [file 12967_2020_2434_MOESM4_ESM.jpg]
