# Supplementary material for: External validation of the European risk assessment tool for chronic cardio-metabolic disorders in a Middle Eastern population
Source: J Transl Med. 2020 Jul 2;18:267. doi: 10.1186/s12967-020-02434-5 (PMC7331242; doi:10.1186/s12967-020-02434-5)
Supplement: Supplementary file 2 — Additional file 2: Table S1: Risk assessment tool regression coefficients for the chronic cardiometabolic disease developed in the Dutch population. [file 12967_2020_2434_MOESM2_ESM.docx]

| Additional Table S1: Risk assessment tool for sex specific logistic regression coefficients for the chronic cardio-metabolic disease developed in the Dutch population*(1) | | | | | |
| --- | --- | --- | --- | --- | --- |
|  | **Men** | **Point scores** |  | **Women** | **Point scores** |
| Age (years) |  |  |  |  |  |
| - <45 | Reference | 0 |  | Reference | 0 |
| - 45-49.9 | 0.91 | 13 |  | 0.69 | 10 |
| - 50-54.9 | 1.20 | 17 |  | 1.08 | 16 |
| - 55-59.9 | 1.57 | 22 |  | 1.54 | 23 |
| - 60-64.9 | 2.34 | 33 |  | 1.98 | 29 |
| - 65-69.9 | 2.66 | 37 |  | 2.55 | 37 |
| - 70-74.9 | 3.26 | 46 |  | 3.34 | 49 |
| - 75-84.9 | 4.29 | 61 |  | 4.06 | 60 |
| BMI (kg/m^2^) |  |  |  |  |  |
| - <25 | Reference | 0 |  | Reference | 0 |
| - 25-29.9 | 0.32 | 4 |  | 0.27 | 4 |
| - ≥30 | 0.87 | 12 |  | 0.52 | 7 |
| WC (cm) |  |  |  |  |  |
| - <94 (men)/<80(women) | Reference | 0 |  | Reference | 0 |
| - 94-101.9(men)/<80-87.9(women) | 0.20 | 3 |  | 0.12 | 2 |
| - ≥102(men)/ ≥88(women) | 0.19 | 3 |  | 0.40 | 6 |
| use of antihypertensive (yes) | 0.74 | 10 |  | 0.75 | 11 |
| current smoking(yes) | 0.63 | 9 |  | 0.61 | 9 |
| Parent and/or sibling with MI or stroke (yes) | 0.09 | 1 |  | 0.26 | 4 |
| Parent and/or sibling with diabetes (yes) | 0.30 | 4 |  | 0.21 | 3 |
| BMI: body mass index; WC: waist circumference  *Developed by Logistic regression using the intercept of -3.497 for men and -3.793 for women  Point scores were defined as the same as the Alssema et.al (1) | | | | | |

**References**

1. Alssema M, Newson RS, Bakker SJ, Stehouwer CD, Heymans MW, Nijpels G, et al. One risk assessment tool for cardiovascular disease, type 2 diabetes, and chronic kidney disease. Diabetes care. 2012;35(4):741-8.
