# Supplementary material for: External validation of the European risk assessment tool for chronic cardio-metabolic disorders in a Middle Eastern population
Source: J Transl Med. 2020 Jul 2;18:267. doi: 10.1186/s12967-020-02434-5 (PMC7331242; doi:10.1186/s12967-020-02434-5)
Supplement: Supplementary file 3 — Additional file 3: Table S2: Comparing baseline characteristics between Respondents and non- respondents: Tehran Lipid and glucose study. [file 12967_2020_2434_MOESM3_ESM.docx]

| Additional Table S2: Comparing baseline characteristics between Respondents and non- respondents: Tehran Lipid and glucose study | | | | | | | |
| --- | --- | --- | --- | --- | --- | --- | --- |
|  | **Men** | | |  | **Women** | | |
|  | **Respondents**  **(N=1310)** | **Non-respondents**  **(N=1378)** | **Difference**  **(95% CI)** |  | **Respondents (N=1960)** | **Non-respondents (N=1339)** | **Difference**  **(95% CI)** |
| Age (years) | 47.1(12.8) | 47.2(14.1) | -0.1(-1.06-0.97) |  | 45.3(11.3) | 45.5(12.7) | -0.2(-0.97-0.68) |
| Body mass index(kg/m^2^) | 26.7(4.0) | 26.3(4.2) | 0.4(0.12-0.76) |  | 28.9(4.6) | 28.9(5.04) | 0.0(-0.31-0.39) |
| Waist circumference (cm) | 94.9(10.4) | 93.4(10.7) | 1.5(0.72-2.36) |  | 91.5(11.6) | 91.3(12.9) | 0.2(-0.73-1.06) |
| Use of antihypertensive medications (yes) | 42(3.21) | 59(4.28) | -1.07(-2.5-0.38) |  | 148(7.6) | 137(10.2) | -2.7(-4.8-(-0.6)) |
| Current smoking (yes) | 406(31.0) | 491(35.6) | -4.6(-9.0-(-0.3)) |  | 110(5.61) | 87(6.5) | -0.9(-2.6-0.8) |
| Family history diabetes (yes) | 440(33.6) | 336(24.4) | 9.2(5.1-13.3) |  | 624(31.8) | 433(32.3) | -0.5(-4.4-3.4) |
| Family history premature CVD (yes) | 231(17.6) | 215(15.6) | 2.0(-1.1-5.1) |  | 392(20.0) | 259(19.3) | 0.6(-2.4-3.7) |
| Data are shown as mean (SD) for continues and number (%) for categorical covariates; SD: standard deviation; CI: confidence interval; CVD: cardiovascular disease; CCD: chronic cardio-metabolic disease; T2DM: Type 2 diabetes; CKD; chronic kidney disease; CVD: cardiovascular disease | | | | | | | |
