# Supplementary material for: External validation of the European risk assessment tool for chronic cardio-metabolic disorders in a Middle Eastern population
Source: J Transl Med. 2020 Jul 2;18:267. doi: 10.1186/s12967-020-02434-5 (PMC7331242; doi:10.1186/s12967-020-02434-5)
Supplement: Supplementary file 5 — Additional file 5: Table S3: The clinical performance of the risk assessment tool for 9-year prediction of chronic cardio-metabolic disorders and each of the separate diseases: : Tehran Lipid and glucose study. T2DM: Type 2 diabetes; CKD; chronic kidney disease; CVD: cardiovascular disease. The validation cut-off point: ≥ 25 for men and ≥ 23 for women. The developed cut-off point: ≥ 35 for men and women. The number of urban population of Tehran aged 28–85 year: 7,214,301 men and 7,223,540 women. Number at risk in Tehran according to the validation cut-off point is 2,904,388 men and 5,823,342 women. Number at risk in Tehran according to the developed cut-off point 1,636,120 men and 1,469,007 women. [file 12967_2020_2434_MOESM5_ESM.docx]

| Additional Table S3: The clinical performance of the risk assessment tool for 9-year prediction of chronic cardio-metabolic disorders and each of the separate diseases : : Tehran Lipid and glucose study | | | | | | | | | | | |
| --- | --- | --- | --- | --- | --- | --- | --- | --- | --- | --- | --- |
|  | **Chronic cardio-metabolic disorders** | |  | **T2DM** | |  | **CKD** | |  | **CVD** | |
|  | **Validation** | **Developed** |  | **Validation** | **Developed** |  | **Validation** | **Developed** |  | **Validation** | **Developed** |
| Men (N=1314) | | | | | | | | | | | |
| Number of events at score category | 350 | 222 |  | 147 | 101 |  | 244 | 167 |  | 90 | 55 |
| Number of high-risk populations | 529 | 298 |  | 529 | 298 |  | 529 | 298 |  | 529 | 298 |
| Sensitivity, % | 59.4 | 37.7 |  | 58.3 | 40.1 |  | 64.5 | 44.2 |  | 75.0 | 45.8 |
| Specificity, % | 75.3 | 89.5 |  | 64.0 | 81.4 |  | 69.5 | 86.0 |  | 63.2 | 79.6 |
|  |  |  |  |  |  |  |  |  |  |  |  |
| Women (N=1962) | | | | | | | | | | | |
| Number of events at score category | 628 | 359 |  | 204 | 117 |  | 558 | 336 |  | 68 | 50 |
| Number of high-risk populations | 779 | 399 |  | 779 | 399 |  | 779 | 399 |  | 779 | 399 |
| Sensitivity, % | 55.8 | 31.9 |  | 64.8 | 37.1 |  | 56.9 | 34.2 |  | 85.0 | 62.5 |
| Specificity, % | 81.1 | 95.0 |  | 64.3 | 82.5 |  | 76.6 | 93.3 |  | 61.5 | 91.1 |
| T2DM: Type 2 diabetes; CKD; chronic kidney disease; CVD: cardiovascular disease  The validation cut-off point: ≥25 for men and ≥23 for women.  The developed cut-off point: ≥35 for men and women.  The number of urban population of Tehran aged 28-85 year: 7,214,301 men and 7,223,540 women.  Number at risk in Tehran according to the validation cut-off point is 2,904,388 men and 5,823,342 women  Number at risk in Tehran according to the developed cut-off point 1,636,120 men and 1,469,007 women | | | | | | | | | | | |
