# Supplementary material for: External validation of the European risk assessment tool for chronic cardio-metabolic disorders in a Middle Eastern population
Source: J Transl Med. 2020 Jul 2;18:267. doi: 10.1186/s12967-020-02434-5 (PMC7331242; doi:10.1186/s12967-020-02434-5)
Supplement: Supplementary file 6 — Additional file 6: Table S4: Model performance for 6-year and 9-year follow-up using the Iranian waist circumference cut-point (≥ 95 cm): Tehran lipid and glucose study. * With 1000 Bootstrapping. The total sample size was 1314 for men (composite outcome = 589, T2DM = 252, CKD = 378, CVD = 120) and 1926 for women (composite outcome = 1125, T2DM = 315, CKD = 981, CVD = 80). AUC: area under the curve; CI confidence interval; HL; Hosmer–Lemeshow test; T2DM: type 2 diabetes; CKD: chronic kidney disease. [file 12967_2020_2434_MOESM6_ESM.docx]

| Additional Table S4: Model performance for 6-year and 9-year follow-up using the Iranian waist circumference cut-point (≥ 95cm): Tehran lipid and glucose study | | | | | |
| --- | --- | --- | --- | --- | --- |
|  | | **Chronic**  **cardio-metabolic disorders** | **T2DM** | **CKD** | **CVD** |
| Men | | | | | |
|  | | | | | |
| AUC (95% CI) * | **Original Follow-up 6y** | 0.72(0.69-0.75) | 0.64(0.61-0.69) | 0.76(0.72-0.79) | 0.73(0.68-0.79) |
|  | **Original Follow-up 9y** | 0.71(0.68-0.74) | 0.66(0.62-0.69) | 0.71(0.68-0.74) | 0.70(0.66-0.75) |
|  |  |  |  |  |  |
| HL test | **Original Follow-up 6y** | 8.7 (p-value=0.37) | 7.39(p-value=0.49) | 14.8(p-value=0.06) | 23.6(p-value=0.003) |
|  | **Original Follow-up 9y** | 14.38(p-value=0.07) | 8.68(p-value=0.37) | 15.45(p-value=0.05) | 17.6(p-value=0.02) |
| Women | | | | | |
|  | | | | | |
| AUC (95% CI) * | **Original Follow-up 6y** | 0.73(0.71-0.75) | 0.7(0.67-0.73) | 0.71(0.68-0.73) | 0.82(0.78-0.86) |
|  | **Original Follow-up 9y** | 0.73(0.71-0.75) | 0.7(0.67-0.73) | 0.70(0.67-0.72) | 0.81(0.77-0.85) |
|  |  |  |  |  |  |
| HL test | **Original Follow-up 6y** | 5.86(p-value=0.66) | 34.8(p-value<0.001) | 12.3(p-value=0.14) | 23.2(p-value=0.003) |
|  | **Original Follow-up 9y** | 6.53(p-value=0.59) | 38.64(p-value<0.001) | 14.2(p-value=0.07) | 11.36(p-value=0.18) |
| * With 1000 Bootstrapping  The total sample size was 1314 for men (composite outcome=589, T2DM=252, CKD=378, CVD=120) and 1926 for women (composite outcome=1125, T2DM=315, CKD=981, CVD=80)  AUC: area under the curve; CI: confidence interval; HL; Hosmer-Lemeshow test; T2DM: type 2 diabetes; CKD: chronic kidney disease; CVD: cardiovascular disease | | | | | |
