# Supplementary material for: External validation of the European risk assessment tool for chronic cardio-metabolic disorders in a Middle Eastern population
Source: J Transl Med. 2020 Jul 2;18:267. doi: 10.1186/s12967-020-02434-5 (PMC7331242; doi:10.1186/s12967-020-02434-5)
Supplement: Supplementary file 7 — Additional file 7: Table S5. Model performance for 6-year and 9-year follow-after adjusting for educational levels: Tehran lipid and glucose study. * With 1000 Bootstrapping. The total sample size was 1314 for men (composite outcome = 589, T2DM = 252, CKD = 378, CVD = 120) and 1926 for women (composite outcome = 1125, T2DM = 315, CKD = 981, CVD = 80). AUC: area under the curve; CI confidence interval; HL; Hosmer–Lemeshow test; T2DM: type 2 diabetes; CKD: chronic kidney disease; CVD: cardiovascular disease. The AUC was estimated on the predictive probability after further adjustment with educational levels. [file 12967_2020_2434_MOESM7_ESM.docx]

| Additional Table S5: Model performance for 6-year and 9-year follow-after adjusting for educational kevels: Tehran lipid and glucose study | | | | | |
| --- | --- | --- | --- | --- | --- |
|  | | **Chronic**  **cardio-metabolic disorders** | **T2DM** | **CKD** | **CVD** |
| Men | | | | | |
|  | | | | | |
| AUC (95% CI) * | **Original Follow-up 6y** | 0.72(0.69-0.75) | 0.66(0.61-0.70) | 0.76(0.72-0.79) | 0.73(0.68-0.73) |
|  | **Original Follow-up 9y** | 0.71(0.69-0.74) | 0.68(0.64-0.71) | 0.71(0.68-0.74) | 0.71(0.66-0.75) |
|  |  |  |  |  |  |
| HL test | **Original Follow-up 6y** | 3.6 (p-value=0.89) | 11.1 (p-value=0.19) | 4.8(p-value=0.78) | 7.5 (p-value=0.48) |
|  | **Original Follow-up 9y** | 14.1 (p-value=0.08) | 10.8 (p-value=0.2) | 9.15 (p-value=0.33) | 17.5 (p-value=0.02) |
| Women | | | | | |
|  | | | | | |
| AUC (95% CI) * | **Original Follow-up 6y** | 0.74(0.72-0.76) | 0.7(0.67-0.73) | 0.71(0.69-0.74) | 0.82(0.78-0.86) |
|  | **Original Follow-up 9y** | 0.73(0.71-0.75) | 0.69(0.66-0.72) | 0.71(0.69-0.74) | 0.81(0.78-0.85) |
|  |  |  |  |  |  |
| HL test | **Original Follow-up 6y** | 3.4 (p-value=0.9) | 32.1 (p-value<0.001) | 5.36(p-value=0.72) | 14.5 (p-value=0.08) |
|  | **Original Follow-up 9y** | 7.7 (p-value=0.46) | 36.6 (p-value<0.001) | 9.9 (p-value=0.27) | 10.8 (p-value=0.21) |
| * With 1000 Bootstrapping  The total sample size was 1314 for men (composite outcome=589, T2DM=252, CKD=378, CVD=120) and 1926 for women (composite outcome=1125, T2DM=315, CKD=981, CVD=80)  AUC: area under the curve; CI: confidence interval; HL; Hosmer-Lemeshow test; T2DM: type 2 diabetes; CKD: chronic kidney disease; CVD: cardiovascular disease  The AUC was estimated on the predictive probability after further adjustment with educational levels. | | | | | |
