# Supplementary material for: External validation of the European risk assessment tool for chronic cardio-metabolic disorders in a Middle Eastern population
Source: J Transl Med. 2020 Jul 2;18:267. doi: 10.1186/s12967-020-02434-5 (PMC7331242; doi:10.1186/s12967-020-02434-5)
Supplement: Supplementary file 8 — Additional file 8: Table S6: Model performance for 6-year and 9-year follow-after adjusting for marital status: Tehran lipid and glucose study. * With 1000 Bootstrapping. The total sample size was 1314 for men (composite outcome = 589, T2DM = 252, CKD = 378, CVD = 120) and 1926 for women (composite outcome = 1125, T2DM = 315, CKD = 981, CVD = 80). AUC: area under the curve; CI confidence interval; HL; Hosmer–Lemeshow test; T2DM: type 2 diabetes; CKD: chronic kidney disease; CVD: cardiovascular disease. The AUC was estimated on the predictive probability after further adjustment with marital status. [file 12967_2020_2434_MOESM8_ESM.docx]

| Additional Table S6: Model performance for 6-year and 9-year follow-after adjusting for marital status: Tehran lipid and glucose study | | | | | | |
| --- | --- | --- | --- | --- | --- | --- |
|  | | **Chronic**  **cardio-metabolic disorders** | **T2DM** | **CKD** | | **CVD** |
| Men | | | | | | |
|  | | | | | | |
| AUC (95% CI) * | **Original Follow-up 6y** | 0.72(0.69-0.76) | 0.65(0.60-0.69) | 0.76(0.72-0.79) | | 0.73(0.67-0.78) |
|  | **Original Follow-up 9y** | 0.72(0.69-0.74) | 0.66(0.62-0.70) | 0.71(0.68-0.74) | | 0.71(0.67-0.76) |
|  |  |  |  |  | |  |
| HL test | **Original Follow-up 6y** | 5.16 (p-value=0.74) | 5.5 (p-value=0.71) | 11.1 (p-value=0.2) | | 15.1 (p-value=0.06) |
|  | **Original Follow-up 9y** | 12.3 (p-value=0.14) | 4 (p-value=0.86) | 13.7 (p-value=0.09) | | 9.2 (p-value=0.32) |
| Women | | | | | | |
|  | | | | | | |
| AUC (95% CI) * | **Original Follow-up 6y** | 0.73(0.71-0.76) | 0.70(0.68-0.73) | | 0.71(0.69-0.74) | 0.82(0.78-0.86) |
|  | **Original Follow-up 9y** | 0.73(0.71-0.75) | 0.69(0.66-0.72) | | 0.71(0.68-0.73) | 0.81(0.77-0.85) |
|  |  |  |  | |  |  |
| HL test | **Original Follow-up 6y** | 4.8 (p-value=0.78) | 33.3 (p-value=0.0001) | | 8.0 (p-value=0.43) | 11.8 (p-value=0.16) |
|  | **Original Follow-up 9y** | 6.8 (p-value=0.56) | 31.7 (p-value=0.0001) | | 6.7 (p-value=0.57) | 14.2 (p-value=0.08) |
| * With 1000 Bootstrapping  The total sample size was 1314 for men (composite outcome=589, T2DM=252, CKD=378, CVD=120) and 1926 for women (composite outcome=1125, T2DM=315, CKD=981, CVD=80)  AUC: area under the curve; CI: confidence interval; HL; Hosmer-Lemeshow test; T2DM: type 2 diabetes; CKD: chronic kidney disease; CVD: cardiovascular disease  The AUC was estimated on the predictive probability after further adjustment with marital status. | | | | | | |
