# Supplementary material for: External validation of the European risk assessment tool for chronic cardio-metabolic disorders in a Middle Eastern population
Source: J Transl Med. 2020 Jul 2;18:267. doi: 10.1186/s12967-020-02434-5 (PMC7331242; doi:10.1186/s12967-020-02434-5)
Supplement: Supplementary file 9 — Additional file 9: Table S7. Model performance for 6-year (n = 4522) and 9-year (n = 4001) after imputation: Tehran lipid and glucose study. * With 1000 Bootstrapping. 6-year follow-up: 1919 for men (composite outcome = 547, T2DM = 313, CKD = 317, CVD = 104) and 2603 for women (composite outcome = 1182, T2DM = 397, CKD = 997, CVD = 67) 1679. 9-year follow-up: for men (composite outcome = 752, T2DM = 235, CKD = 512, CVD = 87) and 1679 for women (composite outcome = 1445, T2DM = 308, CKD = 1259, CVD = 55). AUC: area under the curve; CI confidence interval; HL; Hosmer–Lemeshow test; T2DM: type 2 diabetes; CKD: chronic kidney disease; CVD: cardiovascular disease. [file 12967_2020_2434_MOESM9_ESM.docx]

| Additional Table S7: Model performance for 6-year (n=4522) and 9-year (n=4001) after imputation: Tehran lipid and glucose study | | | | | |
| --- | --- | --- | --- | --- | --- |
|  | | **Chronic**  **cardio-metabolic disorders** | **T2DM** | **CKD** | **CVD** |
| Men | | | | | |
|  | | | | | |
| AUC (95% CI) * | **Original Follow-up 6y** | 0.71(0.69-0.74) | 0.66(0.62-0.69) | 0.73(0.70-0.76) | 0.75(0.71-0.80) |
|  | **Original Follow-up 9y** | 0.70(0.68-0.73) | 0.67(0.63-0.70) | 0.70(0.67-0.72) | 0.74(0.69-0.80) |
|  |  |  |  |  |  |
| HL test | **Original Follow-up 6y** | 4.12 (p-value=0.84) | 11.9(p-value=0.15) | 10.6(p-value=0.22) | 21.1(p-value=0.007) |
|  | **Original Follow-up 9y** | 8.5(p-value=0.39) | 10.4(p-value=0.04) | 20.9(p-value=0.007) | 16.9(p-value=0.03) |
| Women | | | | | |
|  | | | | | |
| AUC (95% CI) * | **Original Follow-up 6y** | 0.72(0.70-0.74) | 0.68(0.65-0.71) | 0.71(0.68-0.73) | 0.84(0.81-0.88) |
|  | **Original Follow-up 9y** | 0.71(0.69-0.73) | 0.68(0.65-0.71) | 0.69(0.67-0.71) | 0.84(0.80-0.88) |
|  |  |  |  |  |  |
| HL test | **Original Follow-up 6y** | 10.22(p-value=0.25) | 36.4(p-value<0.001) | 16.9(p-value=0.03) | 14.3(p-value=0.07) |
|  | **Original Follow-up 9y** | 11.3(p-value=0.19) | 35.9(p-value<0.001) | 11.7(p-value=0.16) | 13.0(p-value=0.11) |
| * With 1000 Bootstrapping  6-year follow-up:1919 for men (composite outcome=547, T2DM=313, CKD=317, CVD=104) and 2603 for women (composite outcome=1182, T2DM=397, CKD=997, CVD=67) 1679  9-year follow-up: for men (composite outcome=752, T2DM=235, CKD=512, CVD=87) and 1679 for women (composite outcome=1445, T2DM=308, CKD=1259, CVD=55)  AUC: area under the curve; CI: confidence interval; HL; Hosmer-Lemeshow test; T2DM: type 2 diabetes; CKD: chronic kidney disease; CVD: cardiovascular disease | | | | | |
