# Supplementary material for: External validation of the European risk assessment tool for chronic cardio-metabolic disorders in a Middle Eastern population
Source: J Transl Med. 2020 Jul 2;18:267. doi: 10.1186/s12967-020-02434-5 (PMC7331242; doi:10.1186/s12967-020-02434-5)
Supplement: Supplementary file 10 — Additional file 10: Table S8: Model performance for 6-year and 9-year using Cox-regression: Tehran lipid and glucose study. * With 1000 Bootstrapping. AUC: area under the curve; CI confidence interval; HL; Hosmer–Lemeshow test; T2DM: type 2 diabetes; CKD: chronic kidney disease; CVD: cardiovascular disease. [file 12967_2020_2434_MOESM10_ESM.docx]

| Additional Table S8: Model performance for 6-year and 9-year using Cox-regression: Tehran lipid and glucose study | | | | | |
| --- | --- | --- | --- | --- | --- |
|  | | **Chronic**  **cardio-metabolic disorders** | **T2DM** | **CKD** | **CVD** |
| Men | | | | | |
|  | | | | | |
| AUC (95% CI) * | **Original Follow-up 6y** | 0.70(0.69-0.72) | 0.69(0.66-0.72) | 0.75(0.74-0.76) | 0.77(0.73-0.82) |
|  | **Original Follow-up 9y** | 0.69(0.67-0.71) | 0.69(0.65-0.73) | 0.71(0.69-0.73) | 0.72(0.69-0.76) |
|  |  |  |  |  |  |
| Calibration test | **Original Follow-up 6y** | 21.7(p-value=0.01) | 13.1(p-value=0.16) | 10.8(p-value=0.28) | 13.8(p-value=0.13) |
|  | **Original Follow-up 9y** | 26.6(p-value=0.001) | 11.2(p-value=0.26) | 26.0(p-value=0.002) | 13.9(p-value=0.12) |
| Women | | | | | |
|  | | | | | |
| AUC (95% CI) * | **Original Follow-up 6y** | 0.65(0.64-0.67) | 0.71(0.67-0.76) | 0.65(0.63-0.67) | 0.84(0.80-0.88) |
|  | **Original Follow-up 9y** | 0.64(0.63-0.65) | 0.70(0.68-0.73) | 0.64(0.62-0.65) | 0.82(0.78-0.86) |
|  |  |  |  |  |  |
| Calibration test | **Original Follow-up 6y** | 36.3(p-value<0.0001) | 15.4(p-value=0.08) | 31.8(p-value=0.0002) | 13.9(p-value=0.13) |
|  | **Original Follow-up 9y** | 36.9(p-value<0.0001) | 17.07(p-value=0.05) | 56.6(p-value<0.0001) | 21.0(p-value=0.01) |
| * With 1000 Bootstrapping  AUC: area under the curve; CI: confidence interval; T2DM: type 2 diabetes; CKD: chronic kidney disease; CVD: cardiovascular disease | | | | | |
